# Negative symptoms of schizophrenia in patients with acute and transient psychotic disorders

**DOI:** 10.1192/j.eurpsy.2021.2121

**Published:** 2021-08-13

**Authors:** G. Aleshkina, M. Pugacheva, L. Bardenshteyn

**Affiliations:** 1 Department Of Psychiatry And Narcology, A. I. Evdokimov Moscow State University of Medicine and Dentistry, Moscow, Russian Federation; 2 Department Of Psychiatry And Narcology, Moscow State University of Medicine and Dentistry named after A.I. Evdokimov, Moscow, Russian Federation

**Keywords:** negative symptoms, schizophrénia, Acute and transient psychotic disorder

## Abstract

**Introduction:**

The ICD-10 acute and transient psychotic disorders (ATPD, F23) without symptoms of schizophrenia are considered predominantly reactive psychotic disorders or affective pathology. However, negative symptoms of schizophrenia may be revealed in some of these cases after the psychotic reduction.

**Objectives:**

To investigate the association between the developmental characteristics of psychosis and the negative symptoms detection after the psychotic reduction of ATPD without symptoms of schizophrenia.

**Methods:**

68 adult inpatients with ATPD without symptoms of schizophrenia (F23.0) were examined. Negative symptoms were assessed with the PANSS negative symptom subscale (PANSS-NSS). The sample was divided into two groups: with PANSS-NSS score>14 (n=12) and with PANSS-NSS score≤14 (n=56), respectively. Clinical-psychopathological, psychometric and statistical methods were applied.

**Results:**

The results of the study are presented in Table 1.

**Table:** 

Table 1. The ATPD developmental features			
**Features**	**The 1** ^**st**^ **group (n=12)**	**The 2** ^**nd**^ **group (n=56)**	**Pearson’s contingency coefficient (C)**
**Males**	7 (58,3%)	37 (66,1%)	0.062
**Females**	5 (41,7%)	19 (33,9%)	0.062
**Mean age of psychotic onset, years (М±m)**	24,9±10,5	30,8±10,2	-
Family history of schizophrenia*	4 (33,3%)	1 (1,8%)	0.418
Poor premorbid social adaptation*	5 (41,7%)	0	0.520
Prodromal functional decline*	9 (75,0%)	4 (7,1%)	0.550
**Prodromal non-psychotic symptoms**	9 (75,0%)	30 (53,6%)	0.163
**Associated acute stress**	4 (33,3%)	27 (48,2%)	0.113

*p<0,001

**Conclusions:**

The probability of negative symptoms detection in ATPD without symptoms of schizophrenia is relatively strongly associated with the family history of schizophrenia, poor premorbid social adaptation and functional decline prior to the psychotic onset.

**Disclosure:**

No significant relationships.

